# Inference of Antibiotic Resistance and Virulence among Diverse Group A *Streptococcus* Strains Using *emm* Sequencing and Multilocus Genotyping Methods

**DOI:** 10.1371/journal.pone.0006897

**Published:** 2009-09-04

**Authors:** David Metzgar, Darcie Baynes, Christian J. Hansen, Erin A. McDonough, Daisy R. Cabrera, Melody M. Ellorin, Patrick J. Blair, Kevin L. Russell, Dennis J. Faix

**Affiliations:** 1 Department of Respiratory Diseases Research, Naval Health Research Center, San Diego, California, United States of America; 2 Department of Defense Global Emerging Infections Surveillance and Response System (DoD-GEIS), Armed Forces Health Surveillance Center, Silver Spring, Maryland, United States of America; St. Petersburg Pasteur Institute, Russian Federation

## Abstract

**Background:**

Group A *Streptococcus pyogenes* (GAS) exhibits a high degree of clinically relevant phenotypic diversity. Strains vary widely in terms of antibiotic resistance (AbR), clinical severity, and transmission rate. Currently, strain identification is achieved by *emm* typing (direct sequencing of the genomic segment coding for the antigenic portion of the M protein) or by multilocus genotyping methods. Phenotype analysis, including critical AbR typing, is generally achieved by much slower and more laborious direct culture-based methods.

**Methodology/Principal Findings:**

We compare genotype identification (by *emm* typing and PCR/ESI-MS) with directly measured phenotypes (AbR and outbreak associations) for 802 clinical isolates of GAS collected from symptomatic patients over a period of 6 years at 10 military facilities in the United States. All independent strain characterization methods are highly correlated. This shows that recombination, horizontal transfer, and other forms of reassortment are rare in GAS insofar as housekeeping genes, primary virulence and antibiotic resistance determinants, and the *emm* gene are concerned. Therefore, genotyping methods offer an efficient way to predict *emm* type and the associated AbR and virulence phenotypes.

**Conclusions/Significance:**

The data presented here, combined with much historical data, suggest that *emm* typing assays and faster molecular methods that infer *emm* type from genomic signatures could be used to efficiently infer critical phenotypic characteristics based on robust genotype: phenotype correlations. This, in turn, would enable faster and better-targeted responses during identified outbreaks of constitutively resistant or particularly virulent *emm* types.

## Introduction

Group A *Streptococcus pyogenes* (GAS) is a common agent of pharyngitis, febrile respiratory infection, and pneumonia. Less frequently, it can cause severe symptoms such as toxic shock syndrome, necrotizing fasciitis, and sterile rheumatic sequelae [1]. Penicillin is effective in treatment and prevention of GAS [2]–[4], but other antibiotics are often required for individuals who are allergic to penicillin [5]. Different strains of GAS differ widely in terms of both AbR profiles and virulence [1].

GAS strains are commonly discriminated by identity of the M protein, a primary antigen. Traditionally this has been done by GAS culture and serological analysis of purified M protein, and can distinguish 81 recognized M types [6]. Currently, this is usually done by comparing the sequence of a portion of the *emm* gene with a database of sequences from previously identified strains ([6]; http://www.cdc.gov/ncidod/biotech/strep/protocol_emm-type.htm). The two methods are highly concordant [6]. Sequence analysis revealed new types, yielding over 200 currently recognized *emm* types [7]. Multilocus sequence typing (MLST) has also been explored, and found to be tightly correlated with *emm* typing [8].

The correlation between *emm* gene identity and the sequence of several unrelated MLST loci suggests a high degree of genomic stability within this bacterial species. This is also supported by previously reported associations between AbR profile and *emm* type (eg, [9]) and specific correlations between virulence and *emm* type (eg, [10]). This supports exploration of rapid genotyping methods as tools for point-of-care identification of strains and inference of antibiotic susceptibility, outbreak potential, and virulence phenotypes.

One rapid, high-throughput GAS genotyping method has been described. The method involves PCR of 6 housekeeping loci, electrospray ionization mass spectrometry analysis (PCR/ESI-MS) of the resulting amplicons to yield nucleotide content data (deduced from mass), and finally, comparison with data from known *emm* types. This method has been used to identify GAS in uncultured specimens from an outbreak in the United States [7]. That study also presented validation/control data collected on 132 previously isolated and cultured GAS strains of 17 different *emm* sequence types from 5 recruit training centers collected in 2002 and 2003 [7]. PCR/ESI-MS yielded single *emm* type identifications in approximately two thirds of the cases, and lists of two or more equally likely *emm* types in the remaining cases (these groups of equally likely *emm* types are hereafter referred to as *emm* groups). Accuracy was extremely high, with all calls either matching (in the case of single *emm* type calls) or including (in the case of *emm* group calls) the sequence-derived *emm* type.

PCR/ESI-MS can be completed in less than 4 hours, and several hundred specimens can be analyzed in a day using a single instrument. Direct *emm* sequencing is significantly more difficult and time-consuming, since it is usually done on cultured isolates to decrease background and yield clean sequencing results—procedures that take 1 or more days to complete. Direct sequencing from original specimens is possible, but has not been thoroughly explored.

We present AbR and PCR/ESI-MS *emm* group data from 802 clinical GAS isolates collected from patients with pharyngitis or other apparent GAS-associated symptoms over a period of 6 years at 10 US military facilities. The majority of these samples are from military recruits in training, a group that is highly susceptible to GAS outbreaks and regularly prophylaxed with long-acting penicillin G (bicillin).

Accuracy of PCR/ESI-MS *emm* grouping identification is addressed by comparison with *emm* sequence data for 387 isolates. We analyze several temporal/spatial clusters of specific *emm* groups in their entirety to judge the potential for inference of specific *emm* type from PCR/ESI-MS *emm* groups using *emm* sequence data from a representative subset of samples. The associations between specific AbR phenotypes and *emm* type (or *emm* group) are statistically analyzed to quantify the degree of nonrandom association between *emm* type and specific AbR phenotypes. Observed correlations are compared to previously reported associations. The relative predictive values of *emm* sequencing and PCR/ESI-MS are discussed.

Outbreaks, rheumatic sequelae, and invasive GAS disease generally involve only a few of the more than 200 known *emm* types of GAS, and specific strains have been recognized as being more virulent, with greater epidemic potential than other strains [10]–[15]. M1 was associated with severe GAS infections and streptococcal toxic shock syndrome during the mid-to-late1980s. The relative dominance of M1 among strains isolated from patients with uncomplicated pharyngitis was correlated with changes in the rate of severe infections over time—that is, severe GAS complications are more common when M1 is common [10]. The opposite was found to be true for M4 and M12, which are associated with mild and uncomplicated pharyngitis [10]. More recently, M3 was implicated in outbreaks of severe disease in both the United States [7], [13], [16] and the United Kingdom [17]. Serotypes M1, 3, 5, 6, 18 and 24 have all been identified as rheumatogenic, or capable of causing acute rheumatic fever (ARF), and it has been suggested that the cyclic nature of severe infection and ARF rates is a reflection of the cyclic circulation of specific rheumatogenic serotypes [1].

While rheumatogenic strains are often distinguished by serotype, it has been noted that the presence of the *speA* scarlet fever toxin gene seems to be closely linked to rheumatogenic characteristics. However, while many isolates of the most notoriously virulent serotypes (M1 and M3) carry *speA*, others do not [18]. Similarly, mucoid colony morphology has been linked to rheumatogenicity, and can be variable within serotypes [10], [13].

During the collection period represented in this study, there were 7 distinct outbreaks of GAS disease, as measured by sudden increases in apparent rate. There were also 3 GAS-associated fatalities. We specifically typed representatives of all outbreaks, as well as all fatal case isolates, to determine if they were generated by strains previously identified as having a high potential for virulence. We also analyzed a representative subset of the identified *emm* types from the isolate collection for *speA* by PCR [19].

## Results and Discussion

### PCR/ESI-MS correlates well with *emm* sequencing

Lists comparing the results of PCR/ESI-MS and *emm* sequencing are shown in Tables 1 and 2. Of 387 sequenced isolates, PCR/ESI-MS *emm* groups included the correct *emm* type in 384 cases (99.2% accuracy). All three discrepancies involved *emm* type 81 in some way, but each was a unique combination of *emm* type and *emm* group. This suggests there may be an error for type 81 in either the PCR/ESI-MS database or the GenBank sequences used to determine *emm* type by the described sequencing method. The precision of PCR/ESI-MS was significantly less than that of direct sequence analysis, since the average *emm* group included several equally likely *emm* types. Imprecision (multiplicity of equally possible *emm* types in each *emm* group) ranged from 1 to 9, with a weighted average of 2.26 *emm* types per *emm* group across the set of 387 sequenced isolates. However, not all of the possible *emm* types were identified among the isolates: only 29 of the 200+ recognized *emm* types were identified in the sample set. Many of these maintained a stable presence at specific sites, demonstrating that specific *emm* types could be inferred across temporally and geographically clustered sets using paired results from a subset that had been tested by both methods.

**Table 1:** Correlations between *emm* groups and *emm* types.

*emm* group		*emm* type (N)	
1	1 (23)	**81 (1)**	
2	2 (10)		
3	3 (66)		
4	4 (16)		
6	6 (1)		
11	11 (13)		
78	78 (1)		
87	87 (4)		
94	94 (2)	**81 (1)**	
5/58	5 (58)	58 (6)	
12/77	12 (15)		
1/4/9/15/18/39/103/110/119	4 (1)	9 (2)	119 (1)
12/77/118	77 (10)	118 (16)	
17/22/47/84	22 (6)		
18/81/109	18 (13)		
22/25/28/75	75 (1)		
22/28	28 (16)		
22/61/81/104	**6 (1)**		
25/75	75 (33)		
29/53/91/101	29 (3)		
36/77/82/85/96/103	85 (1)		
52/93/101/104/116	101 (4)		
6/22/50/62/81/104	6 (22)		
68/75	68 (5)	75 (6)	
73/81/90	73 (6)		
75/78/89	89 (7)		
9/44/61/82	44 (15)	82 (1)	

*emm* types (identified by *emm* gene sequence analysis) associated with each *emm* group (set of equally likely *emm* types, as identified by T-5000 PCR/ESI-MS analysis); Contradictory identifications are shown in bold. All identities are shown as “identity (number identified)” for that specific associative category.

**Table 2:** Inverse relationships between *emm* groups and *emm* types.

*emm* type		*emm* group (N)	
1	1 (23)		
2	2 (10)		
3	3 (66)		
4	1/4/9/15/18/39/103/110/119 (1)	4 (16)	
5	5/58 (58)		
6	6/22/50/62/81/104 (22)	**22/61/81/104 (1)**	6 (1)
9	1/4/9/15/18/39/103/110/119 (2)		
11	11 (13)		
12	12/77 (15)		
18	18/81/109 (13)		
22	17/22/47/84 (6)		
28	22/28 (16)		
29	29/53/91/101 (3)		
44	9/44/61/82 (15)		
58	5/58 (6)		
68	68/75 (5)		
73	73/81/90 (6)		
75	22/25/28/75 (1)	25/75 (33)	68/75 (6)
77	12/77/118 (10)		
78	78 (1)		
81	**1 (1)**	**94 (1)**	
82	9/44/61/82 (1)		
85	36/77/82/85/96/103 (1)		
87	87 (4)		
89	75/78/89 (7)		
94	94 (2)		
101	52/93/101/104/116 (4)		
118	12/77/118 (16)		
119	1/4/9/15/18/39/103/110/119 (1)		

*emm* groups (set of equally likely *emm* types, as identified by T-5000 PCR/ESI-MS analysis) associated with each *emm* type (identified by *emm* gene sequence analysis); Contradictory identifications are shown in bold. All identities are shown as “identity (number identified)” for that specific associative category.

At least one isolate of each *emm* group per year from each site was sequenced. An additional 188 isolates from the same geotemporal clusters were also sequenced, of which 187 confirmed the initial correspondence, yielding a within-cluster consistency of 99.5%. In the single instance of inconsistency, all isolates from the mixed *emm* group were sequenced, and all remaining isolates shared the initially identified *emm* type. In some cases, specific *emm* sequence types were identified across multiple *emm* groups, all of which contained the correct *emm* type (for example, note the three *emm* groups that were identified for *emm* 75 in Table 2). In these cases, no phenotypic, geographic, or temporal correlates were identified, suggesting that the differences resulted from variation in the precision of the PCR/ESI-MS method.

### Rapid genotyping methods can be used to confidently predict both virulence and antibiotic resistance

Tables 3 and 4 show the distribution of AbR phenotypes among the identified *emm* types (Table 3) and PCR/ESI-MS *emm* groups (Table 4). These tables clearly show strong associations between specific resistance phenotypes and specific genotypes. The overall rates of AbR seen in the collection as a whole are shown in Tables 5 and 6, along with the chi-square probability of the observed distribution among *emm* types being random. Statistical significance of individual *emm* type associations with specific antibiotics is also indicated in Tables 3 and 4, for both positive and negative associations. Both unadjusted and alpha adjusted (Bonferroni adjusted) significance levels are indicated to represent relative levels of confidence. The increased ambiguity yielded by PCR/ESI-MS does not significantly decrease predictive values, though it should be noted that this would not necessarily hold up over longer periods of time, when serotypes sharing PCR/ESI-MS *emm* groups could shift in relative dominance. This is because multiple *emm* types represented by each *emm* group do not always share phenotypes. For example, M25 and M75 share a PCR/ESI-MS *emm* group. M25 is susceptible to erythromycin, and M75 is generally resistant. The predictive value of this *emm* group, 25/75, is high in this study, but only because M75 was far more common than the phenotypically opposite M25. PCR/ESI-MS is readily elaborated, and the designers have noted that it would take 12 primer pairs (as opposed to 6, as used here) to completely distinguish all known *emm* types [7]. Furthermore, this technology is amenable to a higher density multiplex format that could allow many of these assays to be combined, thus enabling elaboration without decreased throughput or additive increases in cost.

**Table 3:** Isolate properties by sequenced *emm* type.

*emm* type	*speA*	*speC*	ERY	CLI	TET	LEV	CHL	OFX
	N+(N)	N+(N)	N+(N)	N+(N)	N+(N)	N+(N)	N+(N)	N+(N)
1	13(13)	0(13)	0(23)^a^	0(23)	0(23)	0(23)	1(16)	1(16)
101	0(1)	1(1)	0(4)	0(4)	0(4)	0(4)	–	–
11	0(9)	9(9)	1(13)	2(13)^a^	0(13)	0(13)	0(12)	0(12)
118	0(16)	16(16)	0(16)	0(16)	0(16)	0(16)	0(9)	0(9)
119	0(1)	1(1)	0(1)	0(1)	**1(1)**	0(1)	0(1)	0(1)
12	0(9)	6(9)	2(15)	1(15)	0(15)	0(15)	0(9)	0(9)
18	1(9)	8(9)	0(13)	0(13)	0(13)	0(13)	0(1)	0(1)
2	0(5)	4(5)	0(9)	0(9)	0(9)	0(9)	0(6)	0(6)
22	0(3)	1(3)	1(6)	0(6)	0(6)	0(6)	0(6)	0(6)
28	0(10)	9(10)	0(16)	0(16)	0(16)	0(16)	1(11)	1(11)
29	0(3)	0(3)	0(3)	0(3)	0(3)	0(3)	–	–
3	12(12)	5(12)	0(66)^b^	0(66)	0(66)^b^	2(66)	8(50)^a^	3(50)
4	0(11)	10(11)	2(17)	0(17)	0(17)	0(17)	0(6)	0(6)
44	0(7)	0(9)	1(15)	0(15)	1(15)	0(15)	3(12)^a^	0(12)
5	0(31)	31(31)	1(58)^a^	0(58)	1(58)^a^	0(58)	6(33)^a^	0(33)^a^
58	0(6)	1(6)	**6(6)** ^b^	1(6)	**6(6)** ^b^	0(6)	0(5)	0(5)
6	0(13)	10(13)	1(24)	0(24)	0(24)	3(24)^a^	0(21)	**20(21)** ^b^
68	0(5)	5(5)	0(5)	0(5)	**4(5)** ^b^	0(5)	0(4)	1(4)
73	0(6)	4(6)	0(6)	0(6)	0(6)	0(6)	0(3)	0(3)
75	0(12)	7(12)	**32(40)** ^b^	2(40)	0(40)^a^	0(40)	1(37)	0(37)^a^
77	0(8)	6(8)	0(10)	0(10)	**8(10)** ^b^	0(10)	0(7)	0(7)
78	0(1)	1(1)	0(1)	0(1)	0(1)	0(1)	0(1)	0(1)
81	0(2)	0(2)	0(2)	0(2)	**1(2)**	0(2)	**1(1)** ^b^	0(1)
82	0(1)	1(1)	0(1)	0(1)	0(1)	0(1)	0(1)	0(1)
85	0(1)	0(1)	0(1)	0(1)	0(1)	0(1)	–	–
87	0(4)	3(4)	0(4)	0(4)	0(4)	0(4)	0(1)	0(1)
89	0(6)	6(6)	0(7)	0(7)	0(7)	0(7)	0(4)	0(4)
9	0(2)	1(2)	0(2)	0(2)	**1(2)**	0(2)	–	–
94	0(2)	0(2)	**1(2)**	0(2)	**1(2)**	0(2)	–	–

Observed rates of antibiotic resistance and scarlet fever exotoxin gene carriage for all identified sequence-based *emm* types. N+(N) = Number of Positives (Number Tested). Bold indicates 50% or greater rates of resistance. Positives either carry a particular *spe* gene or show resistance to a particular antibiotic (inclusive of both intermediate and full resistance). ERY, erythromycin; CLI, clindamycin; TET, tetracycline; LEV, levofloxacin; CHL, chloramphenicol; OFX, ofloxacin.^ a^Independently significant specific association, positive or negative. ^b^Significant after alpha (Bonferroni) adjustment for multiple measures (highly significant). *speA*, scarlet fever exotoxin gene A; *speC*, scarlet fever exotoxin gene C. These following results are not shown in the table: 100% of tested isolates (in all cases N>300) were susceptible to penicillin (PEN), vancomycin (VAN), cefepime (CPM), cefotaxime (CTX), ceftriaxone (CTR), linizolid (LNZ), meropenem (MEM), and gatifloxacin (GAT). 311 were tested with quinupristin-dalfopristim (SYN) and one was resistant, six isolates were tested with trovafloxacin (TVA) and 3 were resistant.

**Table 4:** Isolate properties by PCR/ESI-MS *emm* group.

PCR/ESI-MS *emm* group	*speA*	*speC*	ERY	CLI	TET	LEV	CHL	OFX
	N+(N)	N+(N)	N+(N)	N+(N)	N+(N)	N+(N)	N+(N)	N+(N)
1	14(15)	0(15)	0(38)^a^	0(38)	0(38)	0(38)	2(27)	2(27)
1/4/9/15/18/39/103/110/119	–	3(4)	0(6)	0(6)	2(6)^a^	0(6)	0(2)	0(2)
11	0(9)	9(9)	2(16)	3(16)^b^	2(16)	0(16)	0(14)	0(14)
12/77	0(9)	6(9)	5(25)	3(24)^a^	0(25)	0(25)	1(12)	0(12)
12/77/118	0(24)	22(24)	0(64)^b^	0(64)	8(64)^b^	0(64)	0(23)	0(23)
17/22/47/84	0(3)	1(3)	1(10)	0(10)	0(10)	0(10)	0(10)	0(10)
18/81/109	2(10)	9(10)	0(56)^b^	0(56)	0(56)	1(56)	0(2)	0(2)
2	6(6)	5(6)	0(14)	0(14)	0(14)	0(14)	0(7)	0(7)
22/25/28/75	1 (1)	1 (1)	**1(1)**	0(1)	0(1)	0(1)	**1(1)**	0(1)
22/28	0(11)	10(11)	1(26)	0(26)	0(26)	0(26)	1(17)	1(17)
22/61/81/104	0(1)	1(1)	0(1)	0(1)	0(1)	0(1)	–	–
25/75	0(7)	6(7)	**66(73)^b^**	2(72)	0(73)	0(73)	2(70)^a^	1(70)^a^
29/53/91/101	0(3)	0(3)	0/(6)	0(6)	0(6)	0(6)	–	–
3	0(12)	5(12)	0(132)^b^	0(132)	0(132)^a^	3(132)	16(107)^a^	3(106)^a^
36/77/82/85/96/103	0(1)	0(1)	0(1)	0(1)	0(1)	0(1)	–	–
4	0(12)	9(12)	4(27)	1(27)	0(27)	0(27)	0(9)	0(9)
5/58	0(39)	34(39)	11(188)^a^	4(188)	8(188)	0(188)	13(81)^a^	0(81)^b^
52/93/101/104/116	0(1)	1(1)	0(4)	0/(4)	0(4)	0(4)	–	–
6	0(1)	1(1)	**1(1)**	0(1)	0(1)	0(1)	0(1)	**1(1)**
6/22/50/62/81/104	0(11)	8(11)	0(36)^a^	0(36)	0(36)	3(36)^b^	1(34)	**33(34)^b^**
68/75	0(11)	7(11)	0(13)	0(13)	6(13)^b^	0(13)	0(10)	1(10)
73/81/90	0(6)	4(6)	0(8)	1(8)	0(8)	0(8)	0(3)	0(3)
75/78/89	0(6)	6(6)	0(14)	0(14)	0(14)	0(14)	0(4)	0(4)
78	0(1)	1(1)	0/(2)	0(2)	0(2)	0(2)	0(2)	0(2)
87	0(4)	3(4)	0(5)	0(5)	0(5)	0(5)	0(2)	0(2)
9/44/61/82	0(8)	1(8)	1(28)	0(28)	1(28)	0(28)	6(21)^ a^	0(21)
94	0(3)	0(3)	1(3)	0(3)	**2(3)** ^ a^	0(3)	**1(2)**	0(2)

Observed rates of antibiotic resistance and scarlet fever exotoxin gene carriage for all identified PCR-ESI/MS emm groups. N+(N) = Number of Positives (Number Tested). Bold indicates 50% or greater rates of resistance. Positives either carry a particular *spe* gene or show resistance to a particular antibiotic (inclusive of both intermediate and full resistance). ERY, erythromycin; CLI, clindamycin; TET, tetracycline; LEV, levofloxacin; CHL, chloramphenicol; OFX, ofloxacin.^ a^Independently significant specific association, positive or negative. ^b^Significant after alpha (Bonferroni) adjustment for multiple measures (highly significant). *speA*, scarlet fever exotoxin gene A; *speC*, scarlet fever exotoxin gene C. These following results are not shown in the table: 100% of tested isolates (in all cases N>300) were susceptible to penicillin (PEN), vancomycin (VAN), cefepime (CPM), cefotaxime (CTX), ceftriaxone (CTR), linizolid (LNZ), meropenem (MEM), and gatifloxacin (GAT). 311 were tested with quinupristin-dalfopristim (SYN) and one was resistant, six isolates were tested with trovafloxacin (TVA) and 3 were resistant.

**Table 5:** Rates and significance of nonrandom distribution by sequenced *emm* type.

Antibiotic	ERY	CLI	TET	LEV	OFX	CHL
N tested	386	386	386	386	261	261
#resistant^a^	48	6	36	5	26	21
% resistant	12%	1.5%	9.3%	1.3%	10%	8.1%
*p* (Pearson)	<0.001	0.1	<0.001	0.4	<0.001	0.1

Overall rate of antibiotic resistance among all GAS *emm* types, and the significance of nonrandom association between *emm* type and antibiotic resistance.^ a^Includes both intermediate and fully resistant isolates, as per CLSI definitions. *p* values represent the probability that the observed distribution of resistance among the types resulted from a random distribution among the real population. Pearson tests were run using Monte Carlo method; N = 10,000.

ERY, erythromycin; CLI, clindamycin; TET, tetracycline; LEV, levofloxacin; OFX, ofloxacin; CHL, chloramphenicol.

**Table 6:** Rates and significance of nonrandom distribution by PCR/ESI-MS *emm* group.

Antibiotic	ERY	CLI	TET	LEV	OFX	CHL
N tested	798	796	798	798	460	461
#resistant^a^	94	13	28	6	42	44
% resistant	12%	1.6%	3.5%	0.75%	9.1%	9.5%
*p* (Pearson)	<0.0001	0.02	<0.0001	0.2	<0.0001	0.01

Overall rate of antibiotic resistance among all GAS *emm* groups, and the significance of nonrandom association between *emm* group and antibiotic resistance. ^a^Includes both intermediate and fully resistant isolates, as per CLSI definitions. *p* values represent the probability that the observed distribution of resistance among the types resulted from a random distribution among the real population. Pearson tests were run using Monte Carlo method; N = 10,000.

ERY, erythromycin; CLI, clindamycin; TET, tetracycline; LEV, levofloxacin; OFX, ofloxacin; CHL, chloramphenicol.

The data collected here correlate with past observations. For example, we observed erythromycin resistance among isolates of M4, M5, M6, M11, M12, M22, M44, M58, M75, and M94, with the majority of resistance coming from M75. Other surveys of GAS isolates in which both *emm* type and AbR type were analyzed have suggested that macrolide (erythromycin) resistance in GAS is strongly correlated with M4, M6, M12, and M75. M75 was associated with over 50% of the macrolide resistance among diverse US isolates collected between 1994 and 1997 [20], while M6 was found to be associated with macrolide resistance among isolates collected from Pittsburg schoolchildren [21]. Among isolates collected from patients in Belgium, M6 was also the most common source of macrolide resistance, but M2, M12, and M4 were also common [22]. M4, M12, and M75 were all commonly associated with macrolide resistance in Greek isolates [23]. M4 and M75 were associated with 79% of all erythromycin resistance seen among isolates collected from several cities over a decade in Spain, followed closely by M12 [9]. Collectively, this demonstrates a global and temporally robust association between erythromycin resistance and a few specific strains.

Of the 7 noted outbreaks during the surveillance period, 1 (the only one with high rates of pneumonia) was associated with M3 [7], [16], 1 was associated with M118, and the remaining 5 were associated with M5. The listed strains represented the majority of isolations in the respective outbreaks, though other strains (including M18, M77, M101, and M4) were also isolated during outbreak-related collection activities. Two of the M5 outbreaks were associated with some invasive disease, and two of the three noted fatalities during the surveillance period were associated with M5. The remaining fatality was associated with M77. This agrees with previous identifications of M3 and M5 as particularly virulent *emm* types, and demonstrates a strong correlation between *emm* type and virulence. All M1 and M3 isolates tested in this study were *speA* positive, and both types were very common. M5 was *speA* negative.

The *speA* gene has been implicated in invasive phenotypes. It is interesting that the strain of M5 that became common during the second half of the study period was negative by PCR for *speA*, yet was strongly associated with both invasive and epidemic characteristics. This behavior is similar to that of M5 in outbreaks in 1989 [13], and similar to what is expected of the common *speA*-positive strains like M3 and M18 [11], [13], [16]. It is possible that this strain derives its invasive potential from genes other than *speA*, or it is possible that the gene has mutated such that it yields a false negative result for *speA* by PCR but still retains functional expression of the SpeA protein. Correlational analysis of the data suggests the former since most *speA*-negative *emm* types, including M5, are *speC* positive (see Table 3), while most *speA*-positive types are *speC* negative.

Rapid genotyping methods yield GAS strain identifications that are tightly associated with critical clinical characteristics, including antibiotic resistance, virulence and outbreak potential (transmissibility). Such methods, if applied directly to patient specimens at the point of care, have the potential to offer clinically relevant information useful for both determination of treatment and identification of appropriate public health responses. We hope that others will continue to contribute to the associative data sets that will allow inferential use of such technologies, both for this organism and for other important human pathogens.

A methodological shift from culture-based analyses toward less laborious rapid antigen tests is happening quickly. However, these tests do not offer any AbR data for treatment decisions or for tracking resistance, nor do they offer genotype data useful for tracking transmission and virulence patterns. The replacement of traditional culture-based methods with on-site rapid diagnostic methods should be made contingent upon development of equally informative rapid technologies, or combined with archiving programs that retain enough of the original specimen to allow further characterization when necessary. Rapid multilocus genotyping methods preserve the value and power of culture-based methods, while offering much greater speed and throughput.

## Materials and Methods

### Ethics statement

This research has been conducted in compliance with all applicable federal and international regulations governing the protection of human subjects in research (DoD IRB protocol NHRC.2001.0008). This study was based entirely on the use of deidentified isolates provided with minimal demographic data. These isolates were already-existing, cultured from throat swabs during the process of routine clinical diagnostic procedures at recruit training sites. This protocol was reviewed and approved by the NHRC institutional review board and found to be exempt from consent requirements.

### Sample collection

As part of the US military's ongoing GAS surveillance program, 802 samples were collected at 10 military facilities from January 2002 through December 2007. Collection was irregular, based on prevalence of and access to cases of GAS disease. Some sites ceased submission after switching to rapid antigen tests and abandoning culture methods.

Patients reporting to medical clinics with pharyngitis or other apparent GAS-associated conditions were sampled by oropharyngeal swab. Swabs were streaked on blood agar plates using standard methods by on-site clinical laboratories. Positive GAS isolates were resuspended in tryptic soy broth with 15% glycerol, frozen at −70°C, then shipped to the Naval Health Research Center (NHRC) on dry ice for confirmation and testing. Samples were stored at NHRC at −70°C.

Prior to testing, samples were re-streaked on blood agar plates (5% sheep blood in TSA base) (Hardy Diagnostics, Santa Maria, CA), and incubated at 35–37°C with 5–10% CO_2_. After 18–24 hours of incubation, β-hemolytic colonies were chosen, re-streaked for isolation, and tested for Gram stain phenotype, bacitracin sensitivity (A-disk method, Hardy Diagnostics) and positive GAS latex agglutination reaction (Hardy Diagnostics). Confirmed GAS isolates were then archived, and aliquots were tested for AbR and strain type.

### Antibiotic resistance testing

From January 2002 through July 2006, MIC supplemental III Panels (Becton, Dickinson and Co., Franklin Lakes, NJ) were used. For the remaining period, the BD Phoenix test system and the associated *Streptococci* test panel (Becton, Dickinson) were used. Both systems were used per manufacturers' instructions. For both systems, MIC values were converted to the categorical resistance values “resistant,” “intermediate,” or “sensitive” based on the standards set by the National Committee for Clinical Laboratory Standards (NCCLS) (which became the Clinical and Laboratory Standards Institute [CLSI] during the study period) and the two systems were rigorously cross-validated with identical samples to support comparability of the results. Full resistance and intermediate resistance, as defined by NCCLS, were both considered “resistant” for the purposes of presentation and statistical analysis.

### Strain typing

PCR/ESI-MS *emm* groups were generated for all 802 samples. A subset of 387 of these was tested for *emm* type by sequencing. We initially sequenced one isolate of each PCR/ESI-MS *emm* group from each site during each year it appeared at that site. Correlation data were used to generate a translation matrix for inference of specific *emm* types from the PCR/ESI-MS data. One hundred and eighty-eight further isolates were sequenced to estimate consistency within clusters.

### *emm* sequencing

The sequencing procedure was adapted, using optimized cycling conditions and PCR reagents, from a Centers for Disease Control and Prevention protocol (http://www.cdc.gov/ncidod/biotech/strep/protocol_emm-type.htm). Extraction was performed with the QIAamp 96 DNA blood kit (QIAGEN, Valencia, CA). Products were processed with an ABI Prism 3100 sequencer (Applied Biosystems, Foster City, CA). Sequences were analyzed with Lasergene (DNASTAR, Inc., Madison WI) and submitted to the CDC BLAST-*emm* server (http://www.cdc.gov/ncidod/biotech/strep/strepblast.htm) to obtain *emm* type match information.

### PCR/ESI-MS

Grown isolates were diluted 10-fold in the DNA Dilution Buffer provided in the GAS Genotyping Kit (Ibis Biosciences, Inc., Carlsbad, CA) and boiled at 100°C for 10 min. Automated PCR setup was done on the Janus Liquid Handling Station (PerkinElmer, Inc., Waltham, MA). Five microliters of enzyme mix, containing GAS Kit PCR Enzyme Dilution Buffer (Ibis Biosciences), 2.4 U/rxn FastStart Taq DNA Polymerase (Roche, Indianapolis, IN), and 5 µl of template were added to each well of a GAS Genotyping plate (Ibis Biosciences). PCR cycling conditions were as follows: 95°C for 10 min; followed by 8 cycles of 95°C for 30 sec, 48°C for 30 sec with an increase of 0.9°C per cycle, and 72°C for 30 sec; then 37 cycles of 95°C for 15 sec, 56°C for 20 sec, and 72°C for 20 sec. Final extension was performed at 72°C for 2 min followed by a 4°C hold. PCR was performed on a Bio-Rad DNA Engine (Bio-Rad Laboratories, Hercules, CA). Primer design and sequence information is available elsewhere [7]. Post-PCR desalting, mass spectrometer analysis and *emm* type analysis were performed on the Ibis T5000 platform (Ibis Biosciences), as previously described [7].

### Statistical analysis

Accuracy of PCR/ESI-MS inference was measured in terms of the proportion of PCR/ESI-MS *emm* group identities that included the correct *emm* sequence type. Both PCR/ESI-MS *emm* groups and directly sequenced *emm* types were independently analyzed for correlation with AbR measures over the course of study. The nonrandomness of the distribution of each type of AbR across all *emm* types (and groups) was measured by chi-square analysis. The significance of specific genotype:phenotype associations were measured by binomial probability analysis of the observed distributions of resistance for specific genotypes for each type of resistance, with appropriate alpha adjustment for multiple measures across all strain types.

### *spe* testing

Representatives of all identified *emm* types were tested for *speA* and *speC* by nested PCR [19]. DNA amplification was performed as referenced [19], except for the following changes: the reaction volume was reduced from 50 µl to 25 µl, and the annealing temperature was reduced from 57°C for 1 min to 55°C for 45 sec. The reaction volume and cycling conditions were adjusted and optimized for use with the GoTaq Flexi DNA Polymerase and buffer system (Promega BioSciences, San Luis Obispo, CA, USA). Thermal cycling was performed on a Bio-Rad DNA Engine (Bio-Rad).
